# Establishment of a prognosis prediction model for lung squamous cell carcinoma related to PET/CT: basing on immunogenic cell death-related lncRNA

**DOI:** 10.1186/s12890-023-02792-y

**Published:** 2023-12-15

**Authors:** Yu Han, Zhiqiang Dong, Yu Xing, Yingying Zhan, Jinhai Zou, Xiaodong Wang

**Affiliations:** 1 Nuclear medicine, Cangzhou Central Hospital, Cangzhou, China; 2 2nd Department of Hepatobiliary and Pancreatic Surgery, Cangzhou People’s Hospital, Cangzhou, China; 3 Department of Pathology, Zhangjiakou Integrated Traditional Chinese and Western Medicine Hospital, Zhangjiakou, China

**Keywords:** Lung squamous cell carcinoma, Immunogenic cell death, lncRNA, Prognosis

## Abstract

**Background:**

Immunogenic cell death (ICD) stimulates adaptive immunity and holds significant promise in cancer therapy. Nevertheless, the influence of ICD-associated long non-coding RNAs (lncRNAs) on the prognosis of patients with lung squamous cell carcinoma (LUSC) remains unexplored.

**Methods:**

We employed data from the The Cancer Genome Atlas (TCGA)database to identify ICD-related lncRNAs associated with the prognosis of LUSC using univariate Cox regression analysis. Subsequently, we utilized the LOSS regression model to construct a predictive risk model for assessing the prognosis of LUSC patients based on ICD-related lncRNAs. Our study randomly allocated187 TCGA patients into a training group and 184 patients for testing the predictive model. Furthermore, we conducted quantitative polymerase chain reaction (qPCR) analysis on 43 tumor tissues from LUSC patients to evaluate lncRNA expression levelsPearson correlation analysis was utilized to analyze the correlation of risk scores with positron emission tomography/computed tomography (PET/CT) parameters among LUSC patients.

**Results:**

The findings from the univariate Cox regression revealed 16 ICD-associated lncRNAs linked to LUSC prognosis, with 12 of these lncRNAs integrated into our risk model utilizing the LOSS regression. Survival analysis indicated a markedly higher overall survival time among patients in the low-risk group compared to those in the high-risk group. The area under the Receiver operating characteristic (ROC) curve to differentiate high-risk and low-risk patients was 0.688. Additionally, the overall survival rate was superior in the low-risk group compared to the high-risk group. Correlation analysis demonstrated a positive association between the risk score calculated based on the ICD-lncRNA risk model and the maximum standard uptake value (SUVmax) (*r* = 0.427, *P* = 0.0043) as well as metabolic volume (MTV)of PET-CT (*r* = 0.360, *P* = 0.0177) in 43 LUSC patients.

**Conclusion:**

We have successfully developed a risk model founded on ICD-related lncRNAs that proves effective in predicting the overall survival of LUSC patients.

**Supplementary Information:**

The online version contains supplementary material available at 10.1186/s12890-023-02792-y.

## Introduction

Lung cancer ranks among the most prevalent and fatal malignant tumors globally, with over 2.2 million new cases reported worldwide, including more than 800,000 occurrences in China each year. Due to advanced-stage diagnosis, the five-year survival rate for lung cancer patients stands at only 17.6% [1]. Lung squamous cell carcinoma (LUSC) represents one of the predominant pathological subtypes of lung cancer, displaying an increasing incidence trend in the Chinese population in recent years [2, 3]. Notably, early-stage LUSC patients exhibit a higher 5-year survival rate due to slower growth, delayed metastasis, and enhanced opportunities for surgical resection [4, 5]. Nonetheless, a comprehensive understanding of the mechanisms underlying the onset as well as the progression of LUSC at molecular level is lacking. It is imperative to categorize LUSC patients based on prognosis, thereby improving their outcomes and investigating prognosis-affecting indicators. This endeavor holds paramount significance in enhancing the quality of life for LUSC patients and alleviating disease burdens. The induction of tumor cell apoptosis or other programmed cell death to impede tumor growth serves as the foundation for all cancer treatment protocols [6]. While apoptotic tumor cells were conventionally considered non-immunogenic and immune-tolerant [7, 8], studies have demonstrated immunogenic properties, termed immunogenic cell death (ICD), in certain apoptotic tumor cells [9, 10]. ICD denotes a unique form of regulated cell death (RCD) driven by stress [11] and functions to stimulate the immune system, eliciting an immune response [12, 13]. To date, a total of 33 genes, denoted as ICD-related genes, have been identified to be associated with ICD [14]]. Many scholars have utilized these 33 ICD-related genes to construct prognostic models for various malignancies, such as head and neck squamous cell carcinoma [15], lower-grade glioma [16], as well as ovarian cancer [17]. Hence, the ability to induce immunogenic tumor cell death constitutes a pivotal factor influencing treatment effectiveness and tumor prognosis.

Long non-coding RNA (lncRNA) refers to noncoding RNA exceeding 200 nucleotides, playing pivotal roles in diverse biological processes such as dosage compensation, epigenetic regulation, cell cycle modulation, and cell differentiation regulation [18–20]. Previous research has emphasized the relative abundance and high stability of lncRNA in circulation, rendering it more dependable than other well-characterized genes [21, 22]. At the same time, lncRNAs had been found to affect chemotherapy sensitivity [23, 24]. Additionally, lncRNAs have been shown to impact chemotherapy sensitivity and immunotherapy tolerability in lung cancer [25]. Moreover, significant prognostic differences have been observed in lung cancer patients based on distinct lncRNA expression profiles [26, 27]. Notably, several lncRNAs, including Necroptosis-Related lncRNA [27], N6-Methyladenosine (m6A)-Related lncRNA [22], and ferroptosis-related lncRNAs [28] ], have been linked to the clinical outcomes of LUSC. However, the influence of ICD-related lncRNAs on the prognosis of LUSC patients is still largely unexplored.

In our current investigation, we aimed to identify ICD-related lncRNAs possessing independent prognostic value for constructing a risk model.

## Materials and methods

### Data collection and compilation

We retrieved RNA-seq datasets comprising 51 normal or paracancerous tissues and 502 LUSC tumor tissues from The Cancer Genome Atlas (TCGA) database (https://portal.gdc.cancer.gov/). Additionally, we obtained 504 relevant clinical information datasets. These datasets were organized into a matrix file using the Perl programming language. After excluding patients with incomplete clinical information (comprising age, sex, TNM staging, survival status, and survival time), 187 patients were randomly allocated to training cohorts, while 184 patients were assigned to testing cohorts. Furthermore, we sourced 33 ICD-related genes from a previous study [14].

### Identification of ICD-related gene-associated lncRNAs

Utilizing the “limma” package (www.bioconductor.org/packages/release/bioc/html/limma.html) [29] based on the R programming language, we conducted differential expression analysis for lncRNAs and the 33 ICD-related genes. Subsequently, Pearson correlation analysis was employed to investigate the relationship between ICD-related genes and differentially expressed lncRNAs (Filter condition: correlation coefficient = 0.4, P < 0.001). The interaction network of ICD-related genes-lncRNAs was visualized utilizing the “igraph” package (https://igraph.org/).

### Identification of prognosis-associated ICD-related lncRNAs

In the context of the R programming language, univariate and multivariate Cox regression analyses were performed using the “survival” package to identify ICD-related lncRNAs with prognostic value. Subsequently, we utilized “limma,“ “pheatmap(https://www.rdocumentation.org/packages/pheatmap/versions/1.0.12/topics/pheatmap), “reshape2” (https://www.rdocumentation.org/packages/reshape2/versions/1.4.4) and “ggpubr” (https://www.rdocumentation.org/packages/ggpubr/versions/0.6.0) packages to generate expression heatmaps and boxplots for prognostic ICD-related lncRNAs.

### Clustering of prognosis-associated ICD-related lncRNAs

Initially, the “corrplot(https://www.rdocumentation.org/packages/corrplot/versions/0.92) package was used to visualize the correlation among ICD-related lncRNAs. Furthermore, based on the expression profiles of ICD-related lncRNAs associated with LUSC prognosis, we employed the “ConsensusClusterPlus” package (http://www.bioconductor.org/packages/release/bioc/html/ConsensusClusterPlus.html) [30] to conduct a consensus cluster analysis on LUSC samples. We then compared the expression and survival times of ICD-related lncRNAs in different subtypes.

### Construction and validation of risk models for ICD-associated lncRNAs

Using a dataset of 371 LUSC patients with complete information, we conducted Cox regression analysis to construct risk models related to ICD-associated lncRNAs. The risk score calculation formula comprised coefficients derived from the expression levels of multiple lncRNAs. We used univariate and multivariate Cox regression analysis to assess the effect of risk score on the prognosis of LUSC patients. We then divided LUSC patients into the high-risk / low-risk cluster based on the median risk score. The “survivor” package was employed to compare survival differences between these clusters, with the “timeROC” package assessing the prognosticvalue of risk models for LUSC patients.

### Patients and samples

Between January 2020 and June 2022, 43 patients diagnosed with LUSC underwent surgical treatment, with their tumor tissues, paracancerous tissues, and normal lung tissues frozen in liquid nitrogen. The patient cohort was composed by 31 males as well as 12 females (age range: 48–72 years). Tissue differentiation levels were distributed as follows: 16 cases of low, 20 cases of moderate, and 7 cases of high tissue differentiation. Tumor-Node-Metastasis (TNM) staging revealed 17 cases in stage I, 18 cases in stage II, and 8 cases in stage III.

Informed consent was obtained from all subjects, and the present study was approved by the ethics committee of Cangzhou Central Hospital.

### Real time fluorescence quantitative polymerase chain reaction PCR (RT-qPCR)

Total RNA extraction from tissues was carried out utilizing an RNA extraction Kit (RC101-01, Vazyme, China). Subsequently, cDNA synthesis was performed according to the instructions of the PrimeScript RT reagent (RR047A, Taraka, Japan). A 20 ul RT-qPCR system was prepared following the instructions of a qPCR master mix kit (A6001, Promega, USA). The PCR primer sequences for lncRNA are provided in Supplement Table 1.

**Table 1:** Patient demographic of training cohort and test cohort

Demographic	Training cohort (n = 187)	Test cohort (n = 184)	t/χ2	*P*
Age (mean ± SD, years)	67.65 ± 8.42	67.03 ± 8.91	1.431	0.319
Gender (n (%))				
Male	140 (74.87)	131 (71.20)	0.635	0.426
Female	47 (25.13)	53 (28.80)		
TNM stage (n (%))				
I	96 (51.34)	81 (44.02)	2.030	0.566
II	60 (32.08)	67 (36.41)		
III	29 (15.51)	34 (18.48)		
IV	2 (1.07)	2 (1.09)		
Alive (n (%))				
yes	64 (34.22)	81 (44.02)	3.739	0.053
no	123 (65.78)	103 (55.98)		
Survival time (mean ± SD, years)	1.64 ± 1.34	1.53 ± 1.63	0.742	0.458

Note: SD, standard deviation

### Positron emission tomography/computed tomography (PET-CT) examination methods and image analysis

All patients underwent a PET-CT scan (Biograph 16 h, Siemens) before surgery, with intravenous 2’-deoxy-2’-[^18^F] fluoro-D-glucose (^18^F-FDG) (3.70–5.55 MBq/kg) administered. The region of interest was delineated along the edge of the primary lesion of LUSC, and the maximum standard uptake value (SUVmax) and metabolic volume (MTV) of the primary lesion were automatically obtained using the fixed threshold method (SUVmax = 2.5 was the threshold value).

### Statistical analysis

Statistical analysis was conducted using SPSS 20.0 (IBM, USA). Group differences were compared using unpaired t-tests, student’s t-tests, or chi-square tests. Pearson methods were utilized to analyze the correlation of risk scores with SUV_max_ or MTV in 43 LUSC patients. P < 0.05 was considered statistically significant.

## Results

### Expression of ICD-associated genes in LUSC

**Fig. 1:**
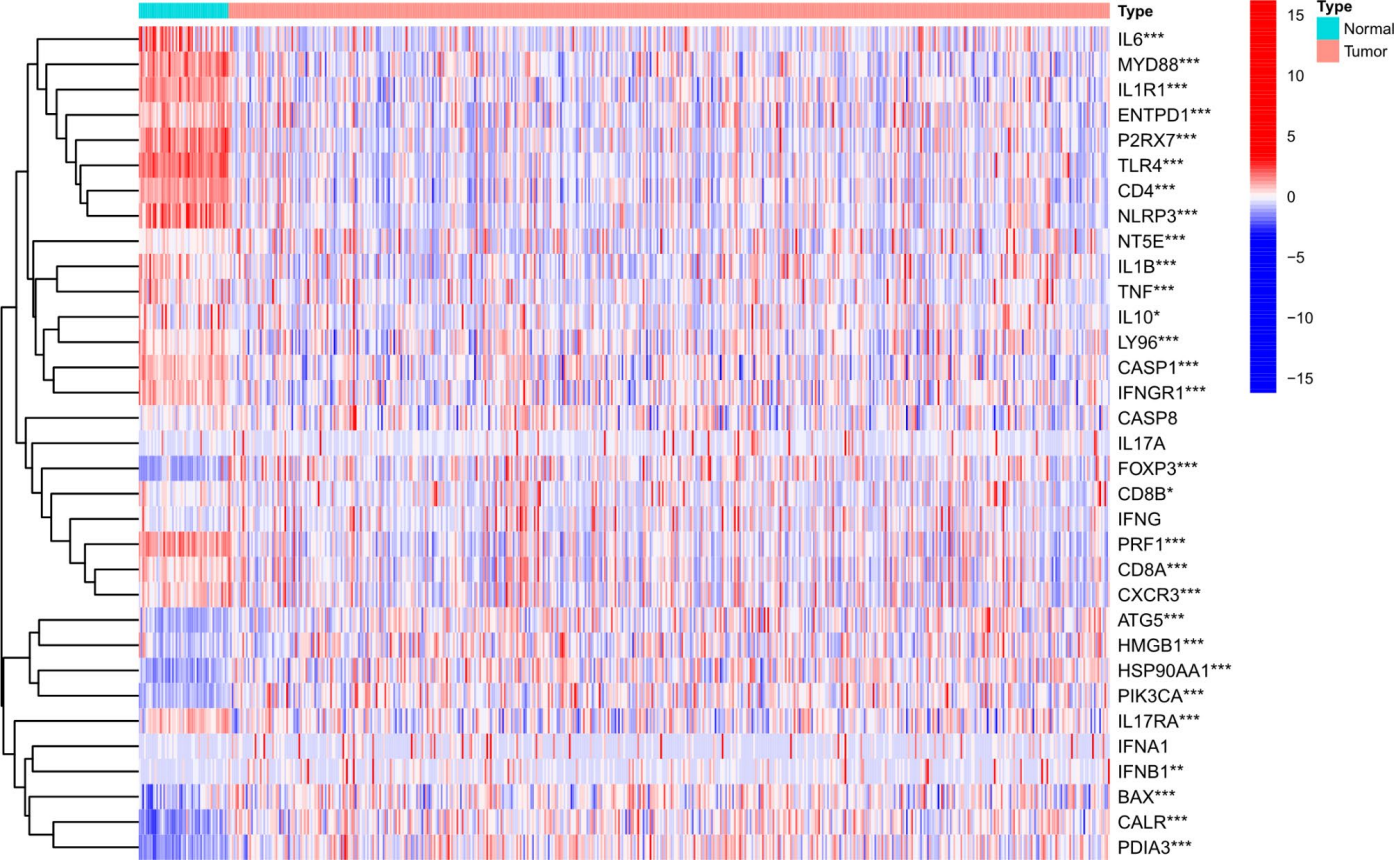
A heat map of the differential expression of 33 immunogenic cell death-related genes between normal tissues and LUSC tissues. *P < 0.05, **P < 0.01, ***P < 0.001

We analyzed 51 normal or paracancerous tissues and 502 LUSC tumor tissues. According to the previous study, we obtained the datasets of 33 ICD-related genes. Subsequently, we found that 10 ICD-related genes (ATG5, BAX, FOXP3, CALR, high mobility group box 1 [HMGB1], heat shock protein [HSP]90AA1, PDIA3, IL-10, NT5E, and PIK3CA) were up-regulated, while 19 genes (CASP1, CD4, CD8A, CD8B, IL-6, LY96, MYD88, NLRP3, CXCR3, ENTPD1, IFNGR1, PRF1, TLR4, ILR1, IL1B, IFNB1, IL17A, and TNF) were down-regulated in LUSC tumor tissues (Fig. 1 and Figure S1).

### Identification of ICD-associated genes-related lncRNAs

**Fig. 2:**
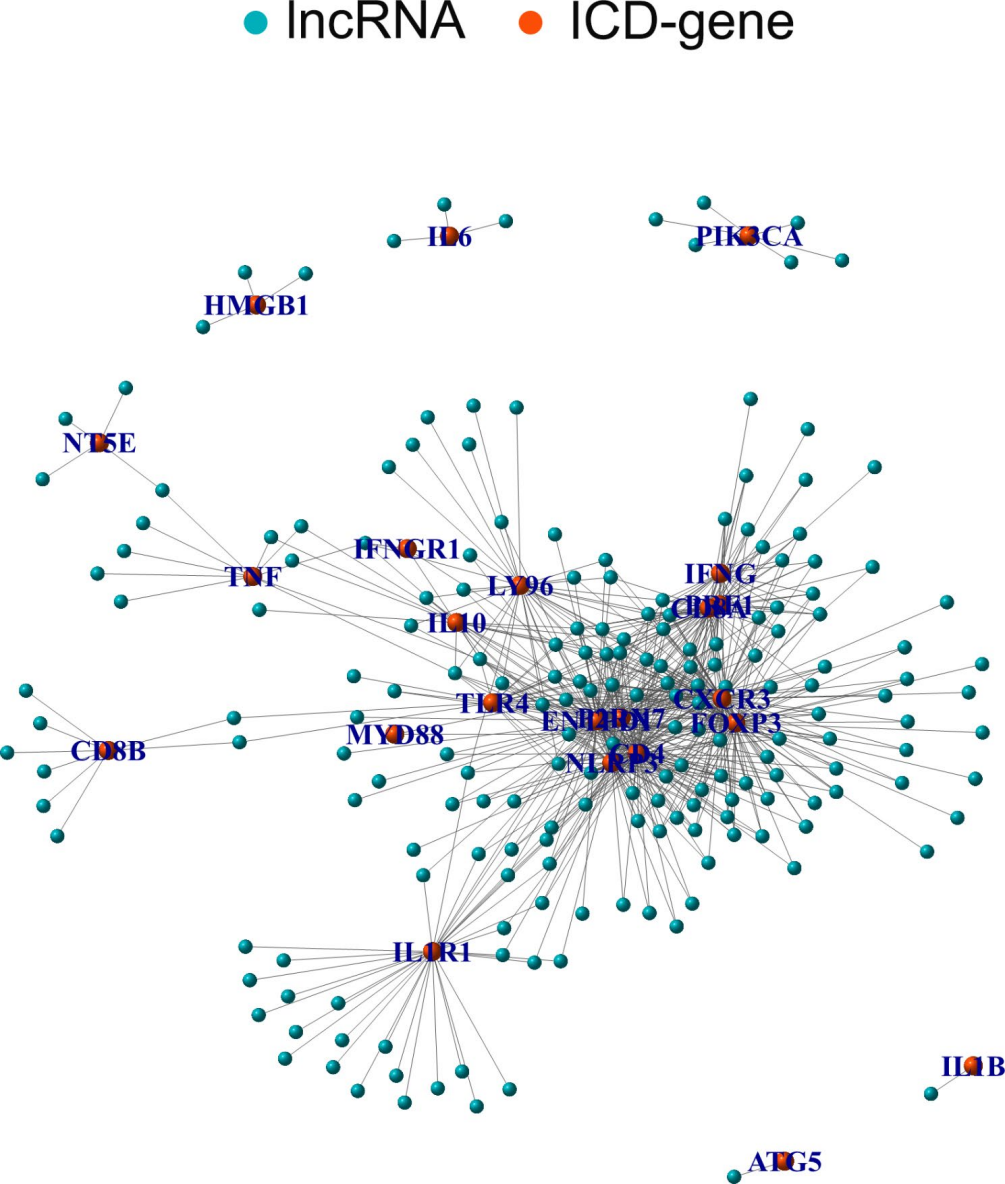
Correlation network of ICD-related genes and related lncRNAs in LUSC.

Pearson correlation analysis was used to assess the relationship between ICD-related genes and differentially expressed lncRNAs, identifying 199 lncRNAs associated with ICD-related genes (Supplement Table 2). Subsequently, the network diagram of ICD-related genes and lncRNA correlation is shown in Fig. 2 (Fig. 2). There were 21 ICD-related genes (CD4, CD8A, CXCR3, ENTPD1, FOXP3, NT5E, PIK3CA, HMGB1, IFNG, IFNGR1, IL-10, IL-1B, P2RX7, PRF1, IL1R1, IL6, LY96, MYD88, NLRP3, TLR4 and TNF) significantly related to various lncRNAs. Among these, CXCR3, ENTPD1, CD4, FOXP3 and NLRP3 were associated with 92, 89, 88, 80 and 60 lncRNAs, respectively; whereas the remaining ICD-related genes were related to fewer lncRNAs (Supplement Table 3).

### Identification of prognosis-associated ICD-related lncRNAs

**Fig. 3:**
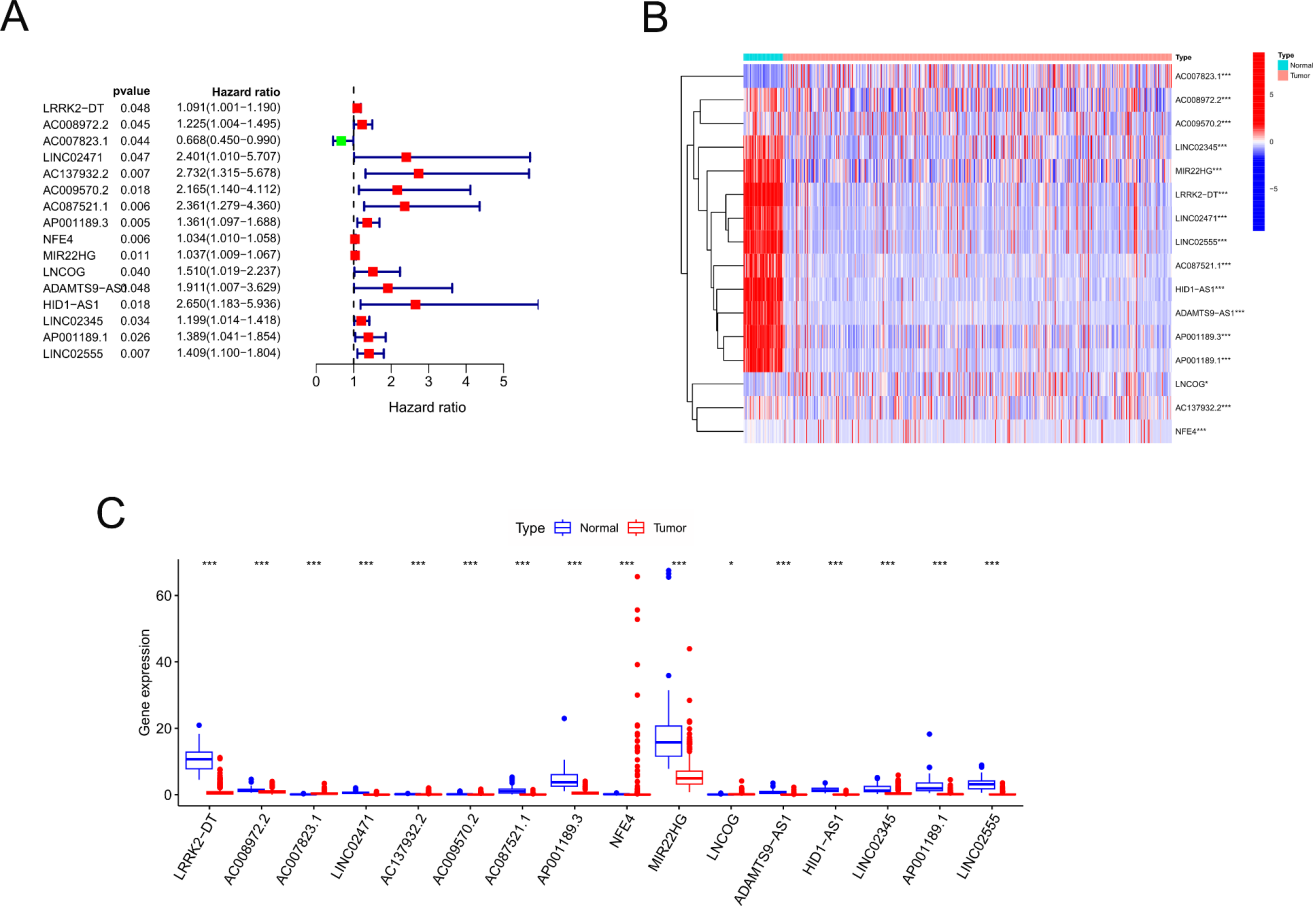
Prognostic analysis of ICD-related lncRNAs. (**A**) Forest map shows ICD-related lncRNAs related to LUSC survival based on univariate Cox regression analysis; (**B**) Expression heat map of ICD-related lncRNAs related to LUSC prognosis between normal normal tissues and LUSC tissues; (**C**) Box plot of ICD-related lncRNAs expression related to LUSC prognosis between normal normal tissues and LUSC tissues *P < 0.05, **P < 0.01, ***P < 0.001

**Fig. 4:**
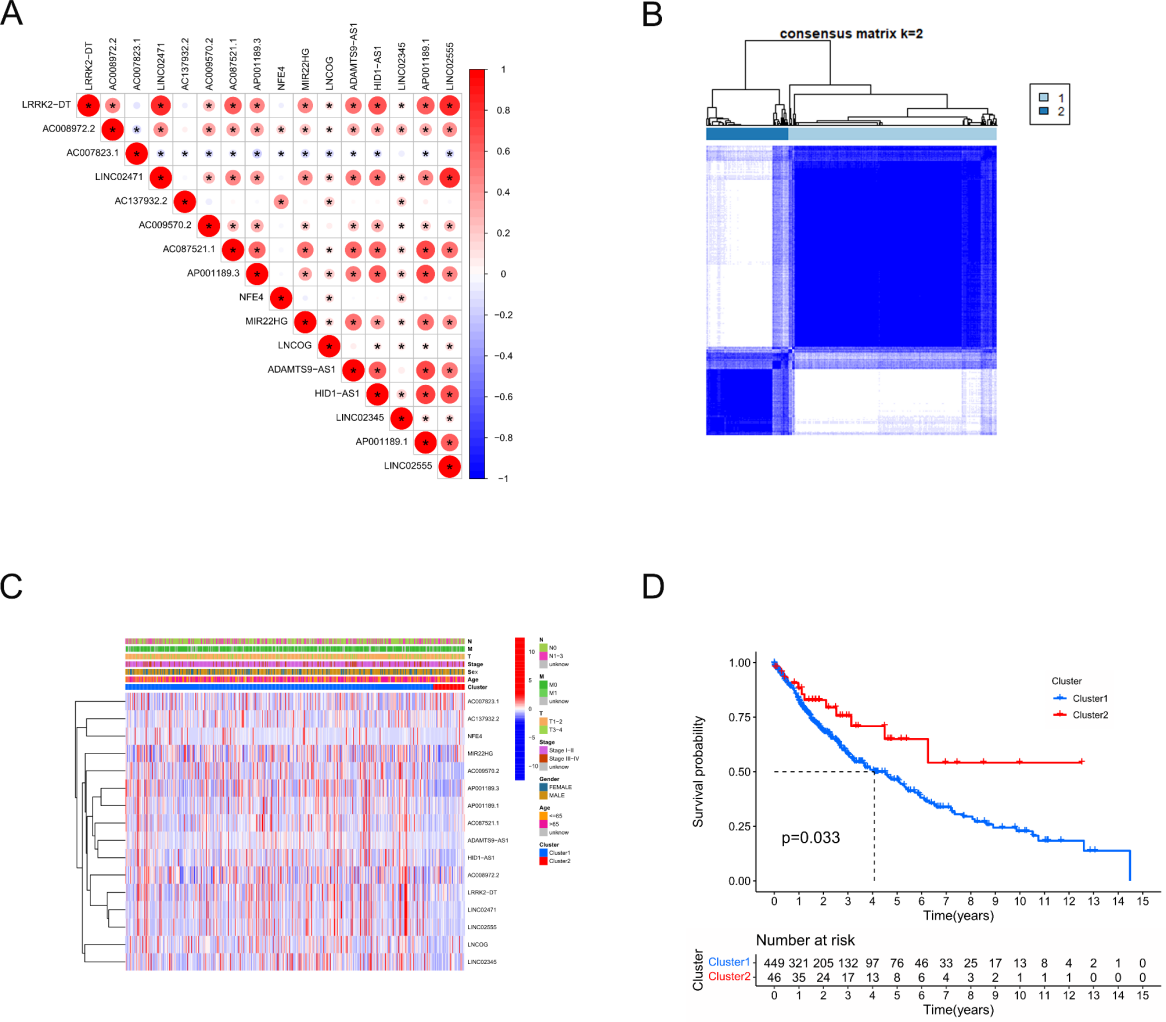
Analysis of clinicopathologic characteristics of LUSC in cluster subgroups; (**A**) Correlation between lncRNAs in ICD-related lncRNAs data set; (**B**) Cluster discrimination was highest for consensus clustering matrix of k = 2; (**C**) Clinicopathologic characteristics and ICD-related lncRNA expression associated with LUSC prognosis between the two clusters; (**D**) Survival analysis in two subgroups of LUSC.

Univariate and multivariate Cox regression analyses identified that a total of 16 ICD-related lncRNAs were associated with the prognosis of LUSC (Fig. 3A). Among these, only three lncRNAs were up-regulated in LUSC tumor tissues (Fig. 3B and C). Next, we analyzed the correlation among these 16 lncRNAs (Fig. 4A)and conducted an unconditional clustering analysis based on 496 RNA-seq data of ICD-related lncRNAs in LUSC patients with complete survival records, resulting in the distinction of two clusters (Fig. 4B and C), where cluster 2 displayed significantly better overall survival than cluster 1 (Fig. 4D).

### Establishing ICD-related lncRNA risk model for LUSC patients

**Fig. 5:**
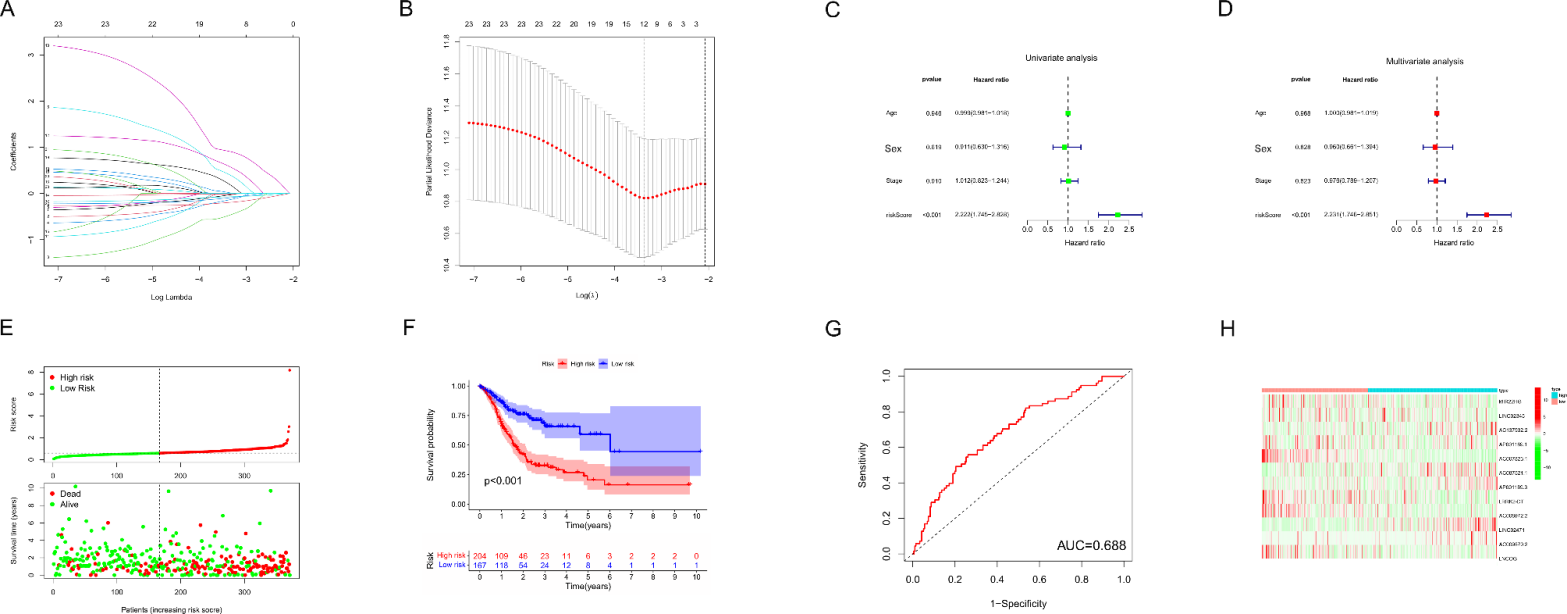
Evaluation of ICD-related lncRNAs risk model in 371 LUSC patients; (**A**) Cross-validation for adjusting parameter selections in the LASSO model; (**B**) LASSO coefficient profiles; (**C-D**) Univariate (**C**) and multivariate (**D**) Cox regression analysis to assess the effect of risk score on the prognosis of LUSC patients; (**E**) Risk scores (up) and survival outcome (down) of each sample; (**F**) Survival analysis of LUSC patients in high risk and low risk sets; (**G**) ROC curve for predicting the prognoses of LUSC patients basing on risk scores; (**H**) Expression of ICD-related lncRNAs in high risk and low risk sets

Using Cox regression analysis on the 16 identified ICD-related lncRNAs, we constructed a risk model consisting of 12 lncRNAs (Fig. 5A and B). The risk score derived from the expression levels of these lncRNAs was determined using coefficients (Supplement Table 4), showing that the risk score independently influenced the prognosis of LUSC patients (Fig. 5C and D). Distribution of risk scores, survival time and the mortality are shown in Fig. 5E. Based on the median risk score, LUSC patients were divided into high-risk and low-risk groups, where the low-risk group exhibited higher total survival time with statistical significance in contrast with the high-risk group (Fig. 5F). The area under the ROC curve for distinguishing high-risk and low-risk patients was 0.688 (Fig. 5G). The expression of ICD-related lncRNAs in high-risk group along with the low-risk group is illustrated in Fig. 5H.

### Validation of ICD-related lncRNA risk model for LUSC patients

**Fig. 6:**
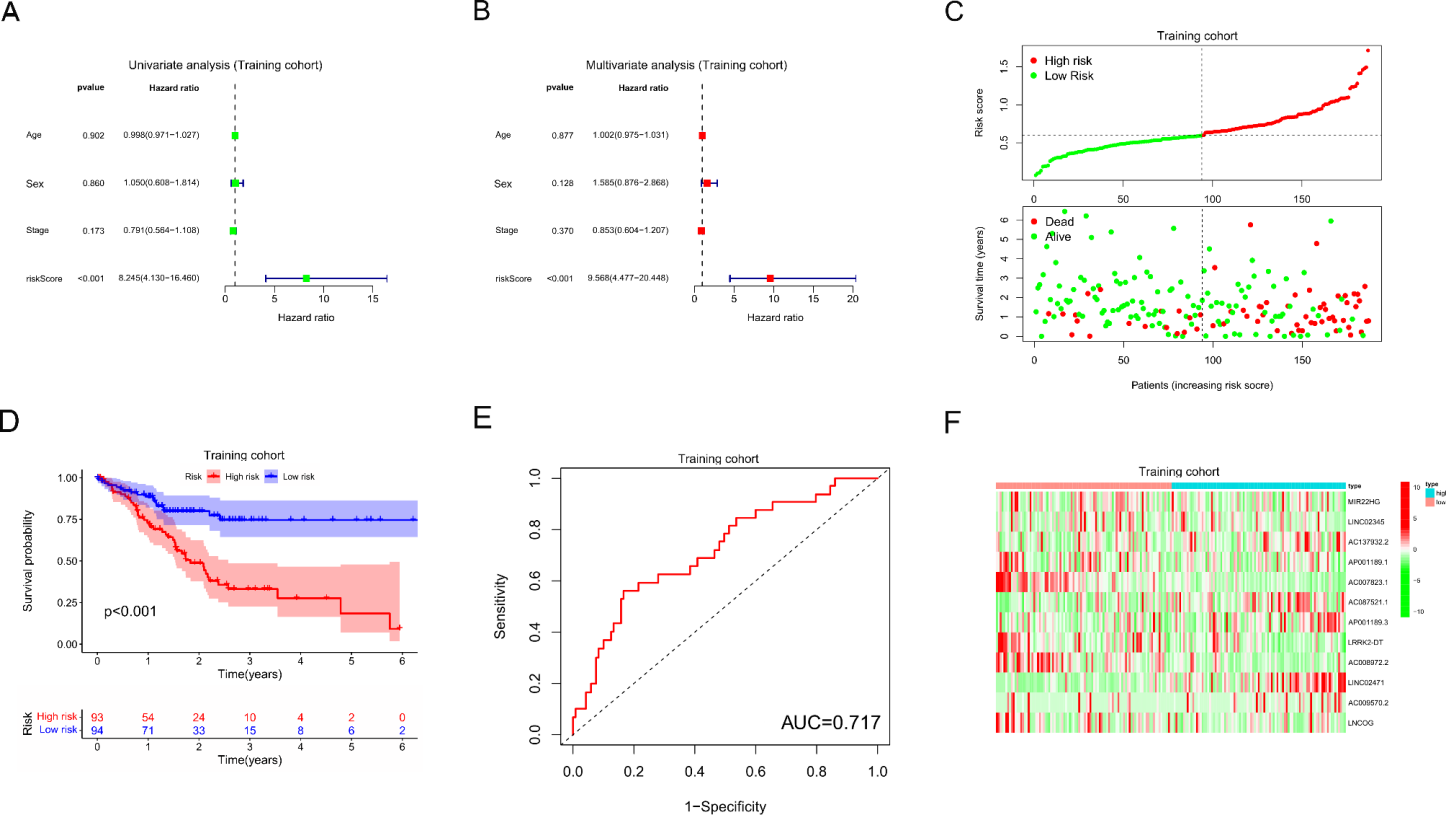
Evaluation of ICD-related lncRNAs risk model in training cohort; (**A-B**) Univariate (**A**) and multivariate (**B**) Cox regression analysis to assess the effect of risk score in training cohort; (**C**) Risk scores (up) and survival outcome (down) of each sample of training cohort; (**D**) Survival analysis of LUSC patients in high risk and low risk sets of training cohort; (**E**) ROC curve for predicting the prognoses of LUSC patients basing on risk scores in training cohort; (**F**) Expression of ICD-related lncRNAs in high risk and low risk sets of training cohort

To verify the accuracy of the risk model in the prognostic prediction of LUSC patients, we used a dataset comprising 371 LUSC patients with complete information. These patients were allocated into training (Supplement Table 5) and test cohorts (Supplement Table 6) exhibiting similar demographic characteristics (Table 1). In the training cohort, univariate and multivariate regression analyses demonstrated that the risk score was an independent influencing factor affecting the prognosis of LUSC patients (Fig. 6A and B). In addition, risk scores, survival time and the status are showed in Fig. 6C. Furthermore, based on the median risk score, LUSC patients were divided into high-risk and low-risk groups. The total survival time of patients in the low- risk group was significantly higher than that in high- risk group (Fig. 6D), and the area under the ROC curve to distinguish high-risk and low-risk patients was 0.688 (Fig. 6E). The expression of ICD-related lncRNAs in high-risk and low-risk sets are showed in Fig. 6F. Fortunately, consistent results were also found in the test cohort (Figure S2).

**Fig. 7:**
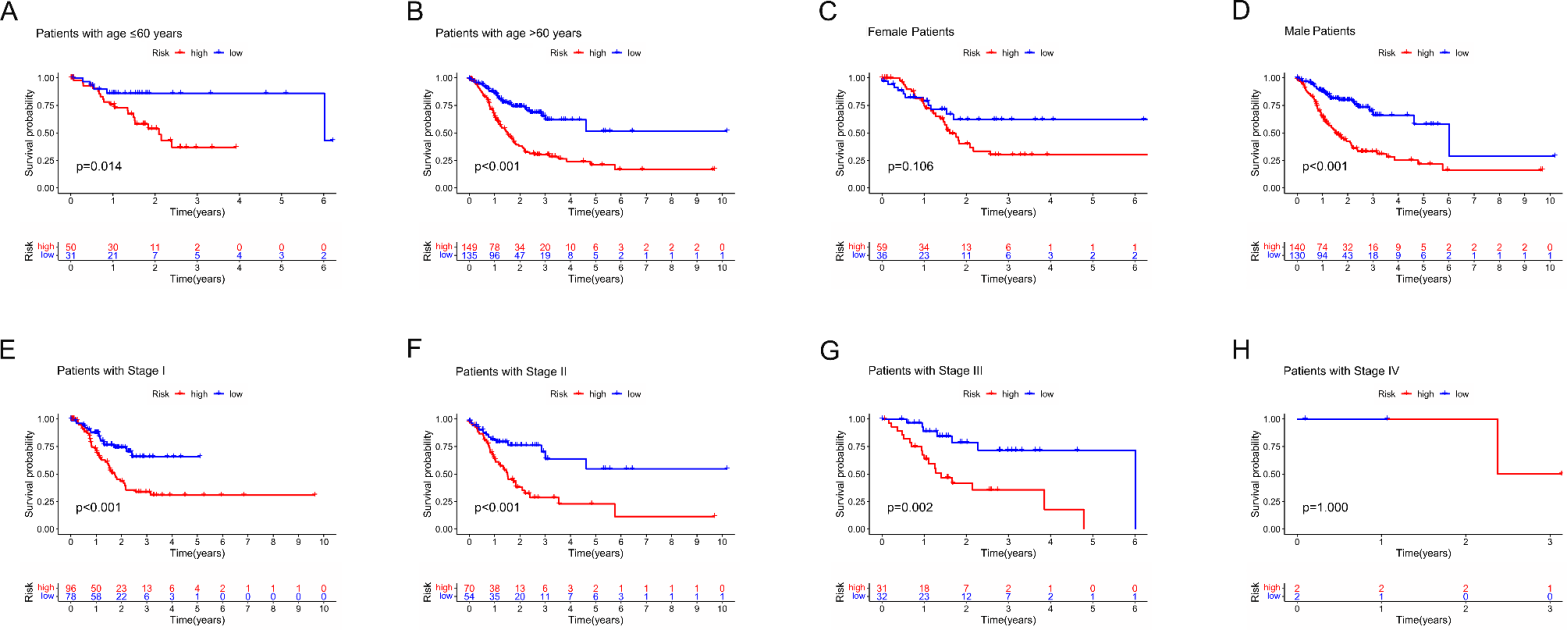
Survival analysis based on risk score in different subtypes of LUSC patients; (**A**) Survival analysis of LUSC patients in high risk and low risk sets of patients with age ≤ 60 years old; (**B**) Survival analysis of LUSC patients in high risk and low risk sets of patients with age > 60 years old; (**C**) Survival analysis of female LUSC patients in high risk and low risk sets of patients; (**D**) Survival analysis of male LUSC patients in high risk and low risk sets of patients; (**E**) Survival analysis of LUSC patients in high risk and low risk sets of patients with stage I; (**F**) Survival analysis of LUSC patients in high risk and low risk sets of patients with stage II; (**G**) Survival analysis of LUSC patients in high risk and low risk sets of patients with stage III; (**H**) Survival analysis of LUSC patients in high risk and low risk sets of patients with stage IV;

In addition, LUSC patients were divided into distinct subtypes based on the age, sex, and clinical TNM stage. In patients In the cohort of low-risk patients stratified by age (≤ 60 years in Fig. 7A, > 60 years in Fig. 7B), gender (female in Fig. 7C, male in Fig. 7D), and disease stage (stage I in Fig. 7E, stage II in Fig. 7F, and stage III in Fig. 7G), the overall survival was higher with statistical significance in comparison with that of high-risk patients. Then, due to the smaller number of patients, there was no significant difference in overall survival between high- and low-risk patients in stage IV patients (Fig. 7H).

### Association of ICD-related lncRNA risk model with ^18^F-FDG PET/CT in LUSC patients

**Fig. 8:**
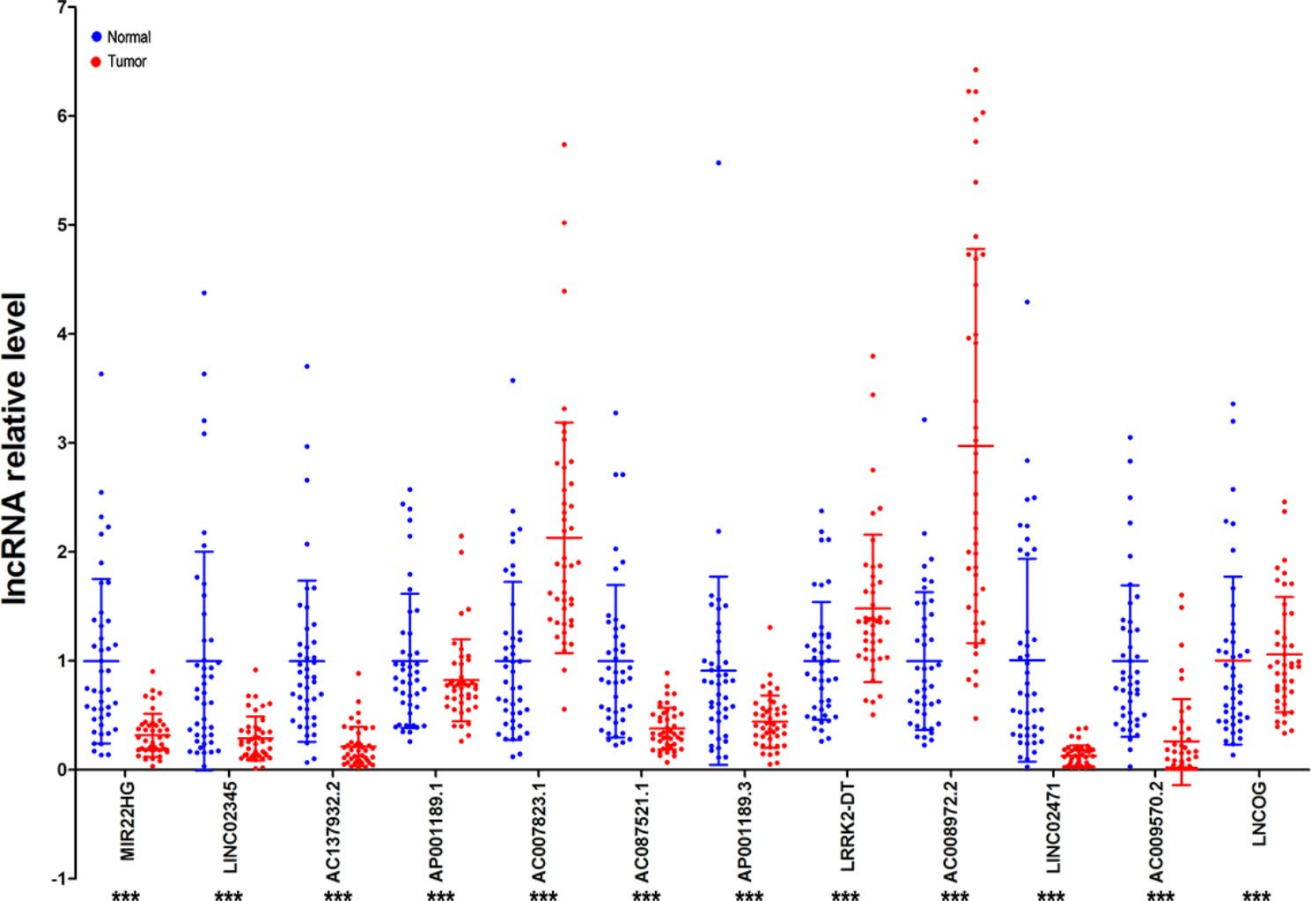
RT-qPCR analysis risk model involved the expression of lncRNAs in tumor tissues and paracancerous normal tissues in 43 LUSC patients

**Fig. 9:**
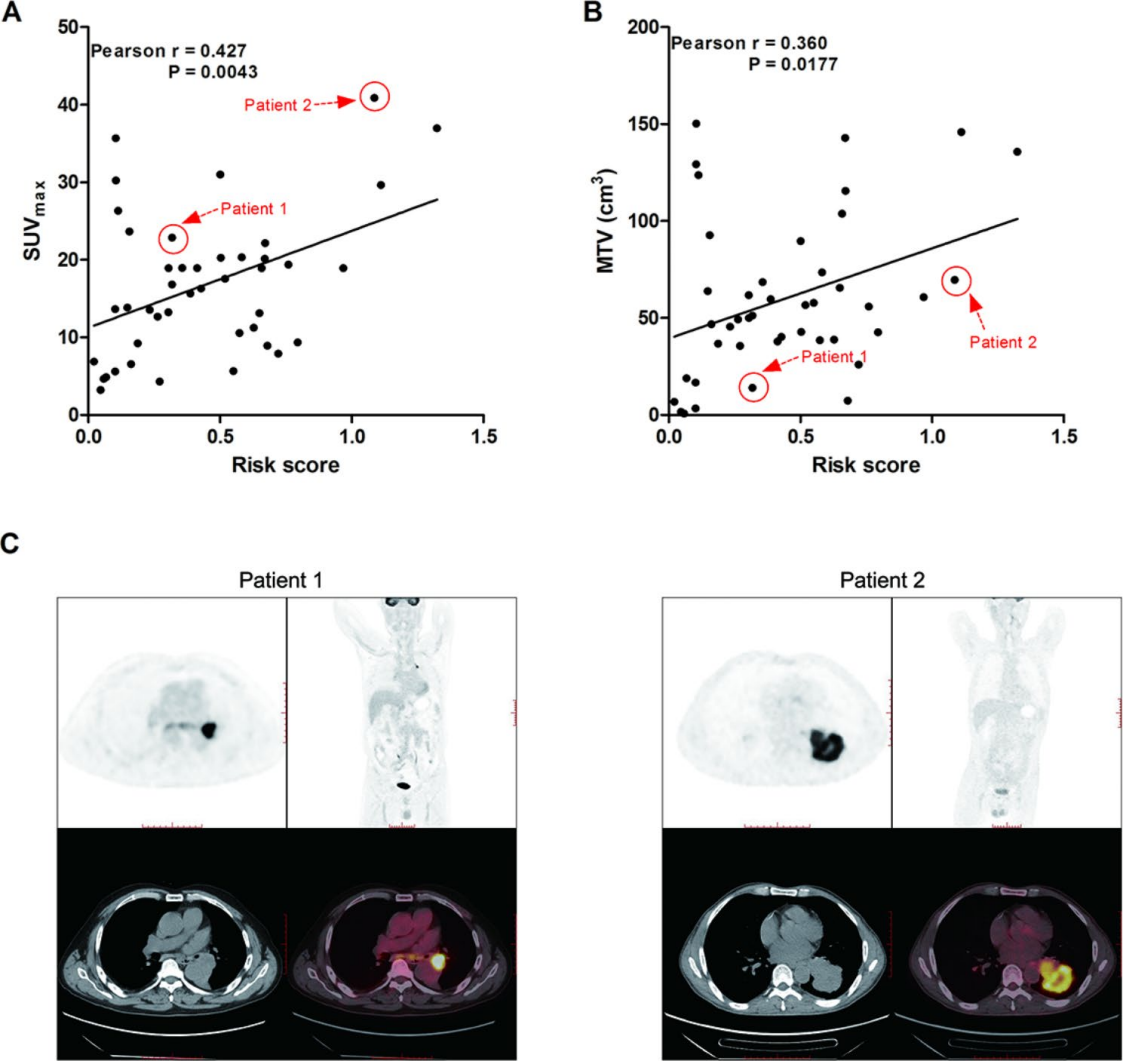
Correlation of risk scores with SUV_max_ or MTV in 43 LUSC patients; (**A**) Correlation scatterplot of risk score and SUV_max_ in 43 LUSC patients; (**B**) Correlation scatterplot of risk score and MTV in 43 LUSC patients; (**C**) PET-CT diagram of two representative LUSC patients

PET-CT is currently an important tool for predicting the prognosis of LUSC patients. We evaluated the correlation between the risk model and PET-CT metabolic parameters using data from 43 LUSC patients who underwent surgical treatment and preoperative PET-CT scans. RT-qPCR analysis revealed significant differences in the expression of the 12 lncRNAs involved in the risk model between LUSC and paracancerous normal tissues (Fig. 8). Additionally, the risk score based on the ICD-lncRNA risk model was positively correlated with SUVmax (*r* = 0.427, *P* = 0.0043) as well as MTV of PET-CT (*r* = 0.360, *P* = 0.0177) (Fig. 9).

## Discussion

Globally, more than 400,000 people have been reported to die from LUSC and its related complications annually [31, 32]. Despite the slow progression of LUSC and its high potential for surgical resection, treatment options are constrained due to its lower responsiveness to radiotherapy and chemotherapy compared to lung adenocarcinoma. Notably, patients with LUSC exhibit distinct clinical features, including smoking history, complications, age, and molecular characteristics, which differ significantly from patients with lung adenocarcinoma, resulting in a poorer prognosis for advanced LUSC patients [5, 33]. Thus, the discovery of novel biomarkers has the potential to guide treatment strategies and aid in the prognostic prediction of LUSC patients.

In this study, we initially categorized LUSC patients into 2 groups using cluster analysis based on 16 ICD-associated lncRNAs linked to LUSC prognosis. We observed notable differences in the cumulative overall survival of these patient types, indicating the potential use of ICD-related lncRNAs as prognostic factors for LUSC patients. Subsequently, we utilized the LOSS regression model to develop a prognostic risk model for LUSC patients, incorporating 12 ICD-related lncRNAs associated with the prognosis of LUSC. Both univariate and multivariate regression analyses, along with total survival analysis and ROC curve evaluations, substantiated the accurate predictive ability of this risk model across different LUSC patient subtypes.

When tumor cells are disrupted by external factors, they can transition from being non-immunogenic to triggering an anti-tumor immune response a phenomenon known as ICD [34, 35]. During ICD, tumor cells release damage-associated molecular patterns (DAMPs) such as calreticulin on the cell surface, secreted HMGB1, ATP molecules, HSP70, and HSP90 [36, 37]. These DAMPs bind to pattern recognition receptors on the surface of dendritic cells, initiating cellular responses that activate both innate and adaptive immunity [38, 39].ICD can be induced by various stressors including intracellular pathogens, traditional chemotherapy drugs, targeted anticancer drugs, and physical therapy [13, 40]. Building upon these findings, our study developed a risk model based on ICD-related lncRNAs associated with the prognosis in LUSC patients. This model stratified LUSC patients into high-risk and low-risk groups, revealing that those within the high-risk category exhibited lower overall survival rates in contrast with the low-risk group.

Currently, PET/CT has been pivotal for the evaluation of the clinical efficacy and prognosis of cancer patients in China post-treatment. The fundamental principle involves illustrating the biological characteristics of tumor cells using various contrast agents to assess cancer cell activity in patients [41, 42]. Comparing PET/CT imaging data of cancer patients before and after treatment in China accurately reflects clinical treatment efficacy, facilitating the early identification of ineffective treatments and predicting post-treatment patient prognosis [43, 44]. However, the high cost and limited availability of equipment/imaging specialists are primary constraints affecting timely PET/CT testing for all cancer patients. In numerous Chinese hospitals, patients often face prolonged waiting periods of weeks for PET/CT tests, with local PET/CT testing costing approximately 5,000–7,000 renminbi (RMB) and whole-body PET/CT testing reaching tens of thousands of RMB, further burdening cancer patients [45, 46].

In the present study, we observed a positive association of the risk score calculated from the ICD-lncRNA model with the SUVmax and MTV obtained from PET-CT. SUVmax, a widely used metabolic parameter in PET-CT imaging, provides a semi-quantitative measure of ^18^F-FDG uptake in tumors, reflecting metabolic activity within highly proliferative tissues [47, 48]. Extensive research has highlighted SUVmax as a vital imaging marker for predicting the prognosis of non-small cell lung cancer patients [46, 49, 50] Notably, it’s essential to recognize that SUVmax only captures the uptake in a segment of the tumor mass and does not account for the entire tumor. Meanwhile, MTV represents the volume of all pixels within a specific range of SUV on PET-CT images, serving as a metabolic parameter based on tumor volume size [51]. Recently, there has been increased recognition of the value of metabolic tumor volume as an indicator of metabolic tumor burden, showing promise as a quantitative PET index [52, 53]. Remarkably, our constructed risk model, requiring only a few hundred RMB and minutes, offers an efficient means to accurately assess patient prognosis compared to PET/CT, which is considerably more costly and time-consuming.

In summary, we identified ICD-related lncRNAs associated with the prognosis of LUSC patients from the TCGA public database and utilized this information to develop a risk model capable of accurately predicting patient prognosis. This risk model not only forecasted patient outcomes in LUSC but also provided guidance for treatment selection. Nevertheless, the limitations of this study should be noted. Specifically, our validation included only 43 LUSC samples, indicating an unavoidable limitation due to the small sample size. Additionally, the dataset of LUSC patients within the TCGA database originated from varied sources, potentially introducing concerns about data heterogeneity given potential differences in data acquisition methodologies across institutions. Lastly, there were potential confounding variables that should be considered.

### Electronic supplementary material

Below is the link to the electronic supplementary material.

Supplementary Material 1

Supplementary Material 2

Supplementary Material 3

Supplementary Material 4

Supplementary Material 5

Supplementary Material 6

Supplementary Material 7

## Data Availability

The datasets generated and/or used during the present study are available from the corresponding author upon reasonable request.
