# Supplementary material for: Establishment of a prognosis prediction model for lung squamous cell carcinoma related to PET/CT: basing on immunogenic cell death-related lncRNA
Source: BMC Pulm Med. 2023 Dec 15;23:511. doi: 10.1186/s12890-023-02792-y (PMC10724919; doi:10.1186/s12890-023-02792-y)
Supplement: Supplementary file 1 — Supplementary Material 1 [file 12890_2023_2792_MOESM1_ESM.docx]

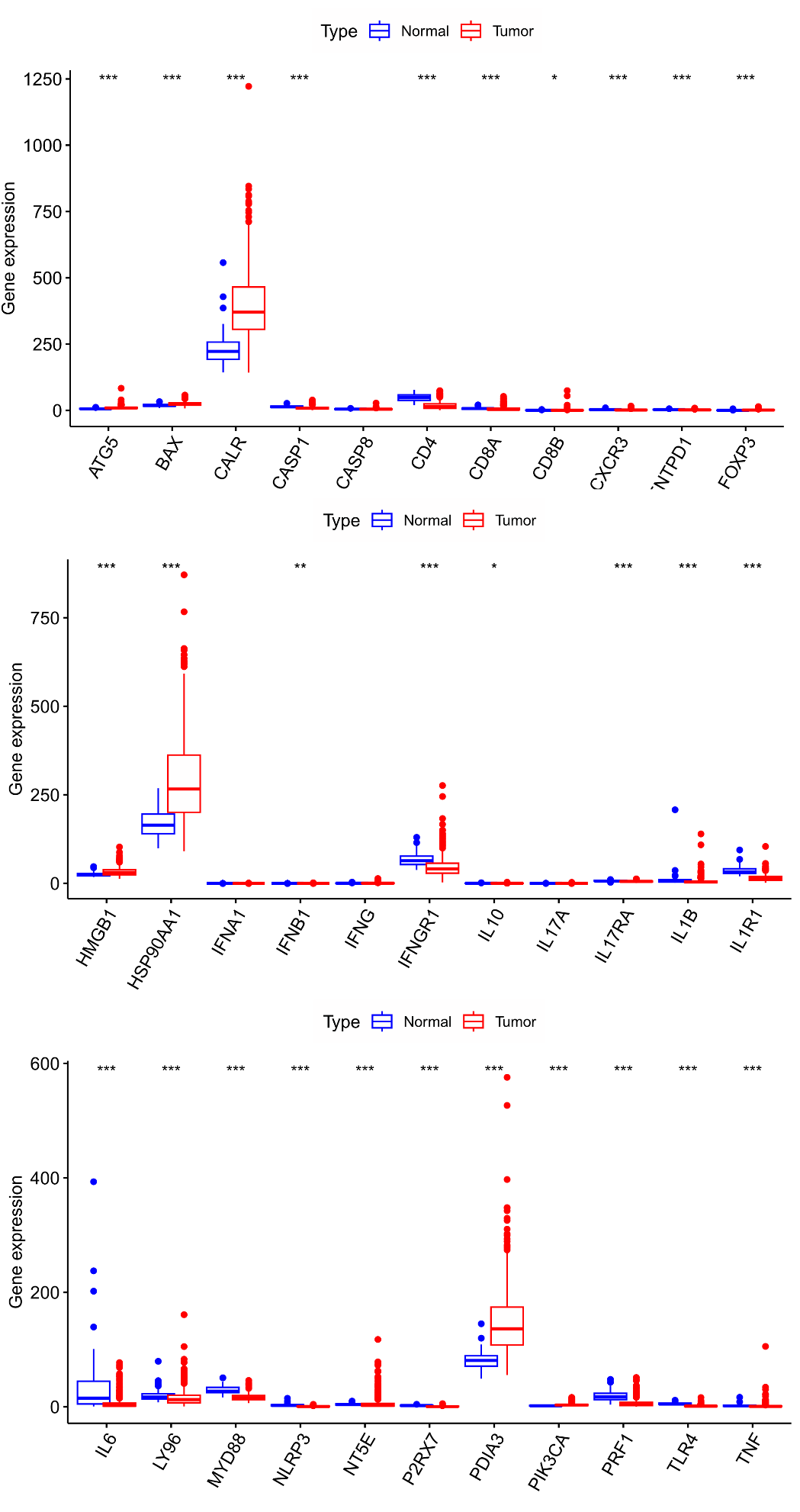


Figure S1 Box plot of the differential expression of 33 immunogenic cell death-related genes between normal tissues and LUSC tissues. *P < 0.05, **P < 0.01, ***P < 0.001.


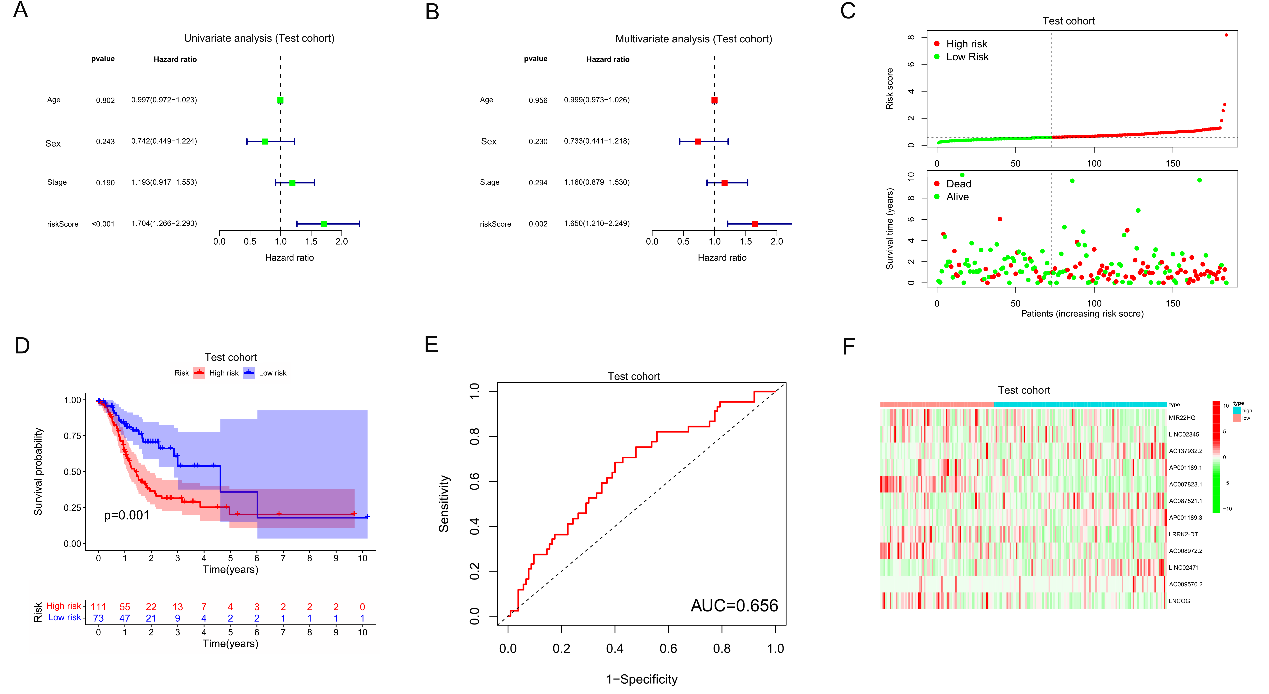


Figure S2 Evaluation of ICD-related lncRNAs risk model in test cohort

(A-B) Univariate (A) and multivariate (B) Cox regression analysis to assess the effect of risk score in test cohort;

(C) Risk scores (up) and survival outcome (down) of each sample of test cohort;

(D) Survival analysis of LUSC patients in high risk and low risk sets of test cohort.

(E) ROC curve for predicting the prognoses of LUSC patients basing on risk scores in test cohort.
