# Supplementary material for: Establishment of a prognosis prediction model for lung squamous cell carcinoma related to PET/CT: basing on immunogenic cell death-related lncRNA
Source: BMC Pulm Med. 2023 Dec 15;23:511. doi: 10.1186/s12890-023-02792-y (PMC10724919; doi:10.1186/s12890-023-02792-y)
Supplement: Supplementary file 2 — Supplementary Material 2 [file 12890_2023_2792_MOESM2_ESM.docx]

Supplement table 1 Primers used in RT-qPCR analysis

| Gene name/ID | Primer sequences |
| --- | --- |
| MIR22HG | Forward: 5'-CCCGACTTCCTTCTTGACCC-3' |
|  | Reverse: 5'-CTTCCTGTAGCCGCTAGGTG-3' |
| LINC02345 | Forward: 5'-CAAGGACTGCACCTTAGCCA-3' |
|  | Reverse: 5'-ATGTGGTTGGAGAAGCCCTC-3' |
| AC137932.2 | Forward: 5'-AAGGTAAACGCAGAGCCGAG-3' |
|  | Reverse: 5'-GACCCTGACAGTCCACGAAG-3' |
| AP001189.1 | Forward: 5'- AGGAAATGACCCTTCGCTGG-3' |
|  | Reverse: 5'-AGGACTTTGATGGTGCCGTT-3' |
| AC007823.1 | Forward: 5'-AGCTGGACACTACTGCAAAAAC-3' |
|  | Reverse: 5'-AGCCTACCCCAATTTGTTGGA-3' |
| AC087521.1 | Forward: 5'-TCGTGTTTGTATCCTCCCTCTG-3' |
|  | Reverse: 5'-TCCAGTGCCTTTGTCTTGGAT-3' |
| AP001189.3 | Forward: 5'- AGGAAATGACCCTTCGCTGG-3' |
|  | Reverse: 5'- AGGACTTTGATGGTGCCGTT-3' |
| LRRK2-DT | Forward: 5'- ATAGAAATCAGGGCGCTGGG-3' |
|  | Reverse: 5'- CGGGTGTCCCAAGTATCAGG-3' |
| AC008972.2 | Forward: 5'- CCAGACCTTGGCTATGGTGAT-3' |
|  | Reverse: 5'-GGATTTTGGGTTTGGAATACCTT-3' |
| LINC02471 | Forward: 5'-CTTTGGACAGCTGCAACAACA-3' |
|  | Reverse: 5'- CCCGGCTCGTGTGAAAAACA-3' |
| \| AC009570.2 \| \| --- \| \|  \| | Forward: 5'- ATGGCCCCACTGTGTAAGGT-3' |
|  | Reverse: 5'- TTTGAGAACACCGTGGCATT-3' |
| LNCOG | Forward: 5'- TGTGATTGTCATTTGGCTGGC-3' |
|  | Reverse: 5'- GGGCCCCACAGTTAGTGTTT-3' |
| β-actin | Forward: 5'-GGCTGTATTCCCCTCCATCG-3' |
|  | Reverse: 5'-CCAGTTGGTAACAATGCCATGT-3' |
